# Integrated host-microbe plasma metagenomics for sepsis diagnosis in a prospective cohort of critically ill adults

**DOI:** 10.1038/s41564-022-01237-2

**Published:** 2022-10-20

**Authors:** Katrina L. Kalantar, Lucile Neyton, Mazin Abdelghany, Eran Mick, Alejandra Jauregui, Saharai Caldera, Paula Hayakawa Serpa, Rajani Ghale, Jack Albright, Aartik Sarma, Alexandra Tsitsiklis, Aleksandra Leligdowicz, Stephanie A. Christenson, Kathleen Liu, Kirsten N. Kangelaris, Carolyn Hendrickson, Pratik Sinha, Antonio Gomez, Norma Neff, Angela Pisco, Sarah B. Doernberg, Joseph L. Derisi, Michael A. Matthay, Carolyn S. Calfee, Charles R. Langelier

**Affiliations:** 1 Chan Zuckerberg Initiative, San Francisco, CA USA; 2 Department of Medicine, Division of Pulmonary, Critical Care, Allergy and Sleep Medicine, University of California San Francisco, San Francisco, CA USA; 3 Department of Medicine, Division of Infectious Diseases, University of California San Francisco, San Francisco, CA USA; 4 Department of Critical Care Medicine, Western University, London, Ontario, Canada; 5 Department of Medicine, Division of Nephrology, University of California San Francisco, San Francisco, CA USA; 6 Department of Medicine, University of California San Francisco, San Francisco, CA USA; 7 Washington University, St Louis, St. Louis, MO USA; 8 Department of Medicine, Zuckerberg San Francisco General Hospital, San Francisco, CA USA; 9 Chan Zuckerberg Biohub, San Francisco, CA USA; 10 Department of Biochemistry and Biophysics, University of California San Francisco, San Francisco, CA USA

**Keywords:** Infectious-disease diagnostics, Diagnostic markers, Molecular medicine, Infectious diseases, Next-generation sequencing

## Abstract

We carried out integrated host and pathogen metagenomic RNA and DNA next generation sequencing (mNGS) of whole blood (*n* = 221) and plasma (*n* = 138) from critically ill patients following hospital admission. We assigned patients into sepsis groups on the basis of clinical and microbiological criteria. From whole-blood gene expression data, we distinguished patients with sepsis from patients with non-infectious systemic inflammatory conditions using a trained bagged support vector machine (bSVM) classifier (area under the receiver operating characteristic curve (AUC) = 0.81 in the training set; AUC = 0.82 in a held-out validation set). Plasma RNA also yielded a transcriptional signature of sepsis with several genes previously reported as sepsis biomarkers, and a bSVM sepsis diagnostic classifier (AUC = 0.97 training set; AUC = 0.77 validation set). Pathogen detection performance of plasma mNGS varied on the basis of pathogen and site of infection. To improve detection of virus, we developed a secondary transcriptomic classifier (AUC = 0.94 training set; AUC = 0.96 validation set). We combined host and microbial features to develop an integrated sepsis diagnostic model that identified 99% of microbiologically confirmed sepsis cases, and predicted sepsis in 74% of suspected and 89% of indeterminate sepsis cases. In summary, we suggest that integrating host transcriptional profiling and broad-range metagenomic pathogen detection from nucleic acid is a promising tool for sepsis diagnosis.

## Abstract (web summary)

Combined host transcriptional profiling and metagenomic pathogen detection of whole blood and plasma samples from critically ill patients enables the generation of classifiers for reliable prediction of sepsis.

## Main

Sepsis causes 20% of all deaths globally and contributes to 20–50% of hospital deaths in the United States alone^1,2^. Early diagnosis and identification of the underlying microbial pathogens is essential for timely and appropriate antibiotic therapy, which is critical for sepsis survival^3,4^. Yet in over 30% of cases, no aetiologic pathogen is identified^5^, reflecting the limitations of current culture-based microbiologic diagnostics^6^. Adding additional complexity is the need to differentiate sepsis effectively from non-infectious systemic illnesses, which often appear clinically similar at the time of hospital admission.

As a result, antibiotic treatment often remains empiric rather than pathogen-targeted, with clinical decision-making based on epidemiological information rather than individual patient data. Similarly, clinicians often continue empiric antimicrobials despite negative microbiologic testing for fear of harming patients in the setting of falsely negative results. Both scenarios lead to antimicrobial overuse and misuse, which contributes to treatment failures, opportunistic infections such as *C. difficile* colitis and the emergence of drug-resistant organisms^7^.

With the introduction of culture-independent methods such as metagenomic next generation sequencing (mNGS), limitations in sepsis diagnostics may be overcome^8,9^. Recent advancements in plasma cell-free DNA sequencing have expanded the scope of metagenomic diagnostics by enabling minimally invasive detection of circulating pathogen nucleic acid originating from diverse anatomical sites of infection^9^. However, the clinical impact of plasma DNA metagenomics has been questioned due to frequent identification of microbes of uncertain clinical relevance, inability to detect RNA viruses that cause pneumonia and limited utility in ruling-out presence of infection^10,11^.

Whole-blood transcriptional profiling offers the potential to mitigate these limitations by capturing host gene expression signatures that distinguish infectious from non-infectious conditions and viral from bacterial infections^12,13^. However, because transcriptional profiling exclusively captures the host response to infection, it does not provide precise taxonomic identification of sepsis pathogens, which limits the utility of this approach when performed alone. Further, transcriptional profiling has traditionally required isolating peripheral blood mononuclear cells, or stabilizing whole blood in specialized collection tubes, and it has remained unknown whether a simple plasma specimen could yield informative data for host-based infectious disease diagnosis.

In recent work, a single-sample metagenomic approach combining host transcriptional profiling with unbiased pathogen detection was developed to improve lower respiratory tract infection diagnosis^14^. Sepsis, defined as ‘life-threatening organ dysfunction from a dysregulated host response to infection^15^’, provides an additional clear use case for this integrated host-microbe metagenomics approach. Here we study a prospective cohort of critically ill adults to develop a sepsis diagnostic assay that combines host transcriptional profiling with broad-range pathogen identification. By applying machine learning to high dimensional mNGS data, we evaluate host and microbial features that distinguish microbiologically confirmed sepsis from non-infectious critical illness. We then demonstrate that plasma nucleic acid can be used to profile both host and microbe for precision sepsis diagnosis.

## Results

### Clinical features of study cohort

**Fig. 1:**
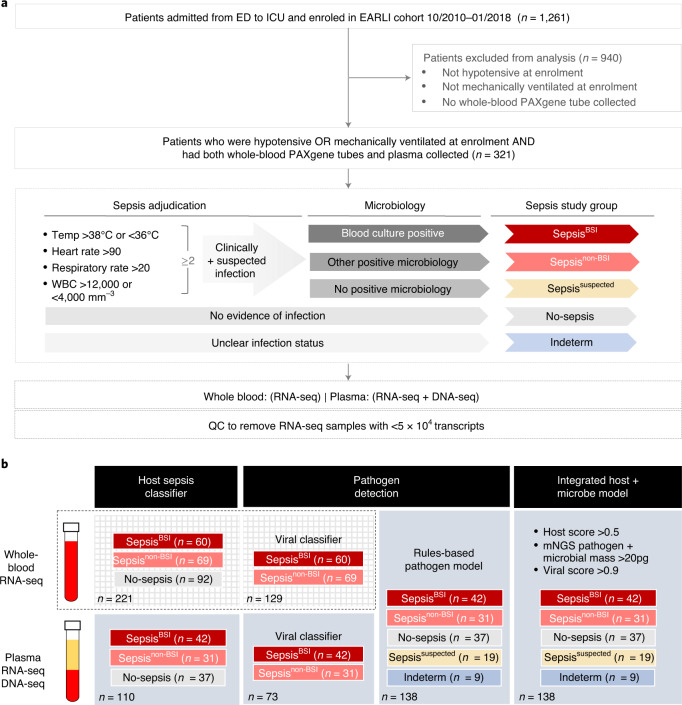
Study overview. **a**, Study flow diagram. Patients studied were enrolled in the EARLI cohort. Sepsis adjudication performed following hospital discharge was based on ≥2 SIRS criteria plus clinical suspicion of infection and was used to delineate five patient subgroups. Following QC, whole blood was subjected to RNA-seq, and plasma to RNA-seq and DNA-seq. WBC, white blood cell count. **b**, Analytic approaches. Host transcriptional sepsis diagnostic classifiers were trained and tested on RNA-seq data from whole blood (*n* = 221) or plasma (*n* = 110), with a goal of differentiating patients with microbiologically confirmed sepsis (Sepsis^BSI^ + Sepsis^non-BSI^) from those without clinical evidence of infection (No-sepsis). Viral infections were identified via a secondary host transcriptomic classifier. Sepsis pathogens were detected from plasma nucleic acid using mNGS followed by an RBM. Finally, an integrated host + microbe model for sepsis diagnosis was developed and evaluated.

We conducted a prospective observational study of critically ill adults admitted from the Emergency Department (ED) to the Intensive Care Unit (ICU) at two tertiary care hospitals (Fig. 1). Patients were categorized into five subgroups on the basis of sepsis status (Methods). These included patients with: (1) clinically adjudicated sepsis and a microbiologically confirmed bacterial bloodstream infection (Sepsis^BSI^), (2) clinically adjudicated sepsis and a microbiologically confirmed non-bloodstream infection (Sepsis^non-BSI^), (3) suspected sepsis with negative clinical microbiologic testing (Sepsis^suspected^), (4) patients with no evidence of sepsis and a clear alternative explanation for their critical illness (No-sepsis) or (5) patients of indeterminate status (Indeterm). The most common diagnoses in the No-sepsis group were cardiac arrest, overdose/poisoning, heart failure exacerbation and pulmonary embolism. The majority of patients, regardless of subgroup, required mechanical ventilation and vasopressor support (Supplementary Tables 1a,b). Patients with microbiologically proven sepsis (Sepsis^BSI^ + Sepsis^non-BSI^) did not differ from No-sepsis patients in terms of age, gender, race, ethnicity, immunocompromise, APACHEIII score, maximum white blood cell count, intubation status or 28 d mortality. All but one patient (in the No-sepsis group) exhibited ≥2 systemic inflammatory response syndrome (SIRS) criteria^16^.

### Host transcriptional signature of sepsis from whole blood

**Fig. 2:**
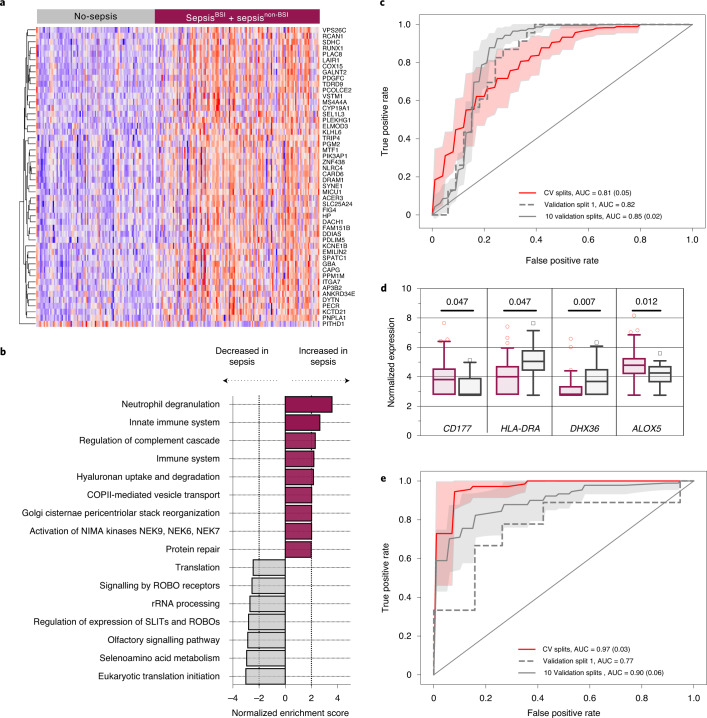
Host gene expression differentiates patients with sepsis from those with non-infectious critical illnesses. **a**, Heat map of top 50 differentially expressed genes from whole-blood transcriptomics comparing patients with microbiologically confirmed sepsis (Sepsis^BSI^ + Sepsis^non-BSI^) versus those without evidence of infection (No-sepsis). The heatmap colour range represents the row *Z*-score of the normalized gene expression values ranging from +4 (red) to −4 (blue). **b**, GSEA of the differentially expressed genes demonstrating pathways enriched in patients with sepsis. Source data including enriched genes and pathway *P* values (hypergeometric test) are provided in Supplementary Data 2a and in the Source Data file. **c**, ROC curve demonstrating performance of the bSVM classifier for sepsis diagnosis from whole-blood transcriptomics (*n* = 221). The AUC and s.d. (in parentheses, when applicable) are listed for cross validation (CV) in the training set (red line: average over 10 random splits; red shaded area: ±1 s.d.), the held-out validation set (dashed grey line) and over 10 randomly generated validation sets (solid grey line: average; grey shaded area: ±1 s.d.). **d**, Plasma RNA-seq expression differences of selected differentially expressed genes previously identified as sepsis biomarkers, with Sepsis patients in maroon (*n* = 73) and No-sepsis patients in grey (*n* = 37). Adjusted *P* values (Benjamini–Hochberg method) from DESeq2 noted above boxplot. Expression data are presented as boxes extending from the 25th to the 75th percentiles, with whiskers extending to the 5th and 95th percentiles, and a central horizontal line at the median. Source data are provided in the Source Data file. **e**, ROC curve demonstrating performance of the bSVM classifier for sepsis diagnosis from plasma RNA (*n* = 110). The AUC and s.d. are listed for CV in the training set (red line: average over 10 random splits; red shaded area: average ± 1 s.d.), the held-out validation set (dashed grey line) and over 10 randomly generated validation sets (solid grey line: average; grey shaded area: average ± 1 s.d.). Source data

We first assessed transcriptional differences between patients with clinically and microbiologically confirmed sepsis (Sepsis^BSI^, Sepsis^non-BSI^) versus those without evidence of infection (No-sepsis) by performing RNA sequencing (RNA-seq) on whole blood specimens (*n* = 221 total) to obtain a median of 5.8 × 10^7^ (95% CI 5.3 × 10^7^–6.3 × 10^7^) reads per sample. Differentially expressed (DE) genes were identified (5,807) at an adjusted *P* < 0.1 (Fig. 2a and Supplementary Data 1). Gene set enrichment analysis (GSEA), a method that identifies groups of genes within a dataset sharing common biological functions^17^, demonstrated upregulation of genes related to neutrophil degranulation and innate immune signalling in the patients with sepsis, with concomitant downregulation of pathways related to translation and ribosomal RNA processing (Fig. 2b and Supplementary Data 2a).

To further characterize differences between sepsis patients with bloodstream versus peripheral site (for example, respiratory, urinary tract) infections, we performed differential gene expression (DE) analysis between the Sepsis^BSI^ and Sepsis^non-BSI^ groups, which identified 5,227 genes (Supplementary Data 3). GSEA demonstrated enrichment in genes related to CD28 signalling, immunoregulatory interactions between lymphoid and non-lymphoid cells, and other functions in the Sepsis^non-BSI^ patients, while the Sepsis^BSI^ group was characterized by enrichment in genes related to antimicrobial peptides, defensins, G alpha signalling and other pathways (Supplementary Data 4).

### Host transcriptional classifier for sepsis diagnosis from whole blood

Given the practical necessity to identify sepsis in both Sepsis^BSI^ and Sepsis^non-BSI^ patients, we constructed a ‘universal’ sepsis diagnostic classifier on the basis of whole-blood gene expression signatures. After dividing the cohort (*n* = 221) into independent training (75% of data, *n* = 165) and validation (25% of data, *n* = 56) groups, we employed a bagged support vector machine (bSVM) learning approach to select genes that most effectively distinguished patients with sepsis (Sepsis^BSI^ and Sepsis^non-BSI^) from those without (No-sepsis). We elected to use a bSVM model due to better performance compared with random forest and gradient boosted trees, which were also tested (Supplementary Table 2). The bSVM model achieved an average cross-validation AUC (area under the receiver operating characteristic (ROC) curve) of 0.81 (s.d. 0.05) over 10 random splits within the training dataset (75% of data, *n* = 165). In the held-out validation set (25% of data, *n* = 56), an AUC of 0.82 was obtained. Additionally, an AUC of 0.85 (s.d. 0.02) was obtained over 10 randomly generated validation sets (Fig. 2c and Supplementary Data 5).

### Host transcriptional classifier for sepsis diagnosis from plasma RNA

Sequencing of plasma DNA has emerged as a preferred strategy for culture-independent detection of bacterial pathogens in the bloodstream^9^. It remains unknown, however, whether plasma RNA could provide meaningful and biologically relevant gene expression data, as sepsis transcriptional profiling studies have historically relied on isolation of peripheral blood mononuclear cells or collection of whole blood.

**Extended Data Fig. 1:**
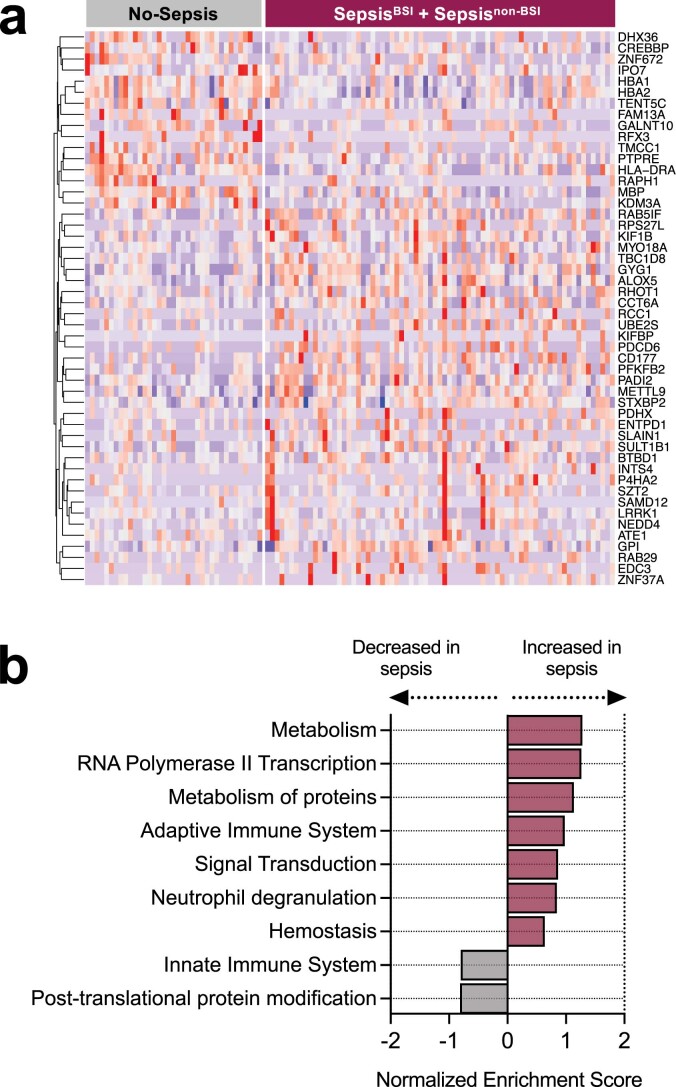
Plasma host gene expression differentiates patients with sepsis from those with non-infectious critical illnesses. Plasma host gene expression differentiates patients with sepsis from those with non-infectious critical illnesses. (**a**) Heatmap of top 50 differentially expressed genes from whole blood transcriptomics comparing patients with microbiologically confirmed sepsis (Sepsis^BSI^ + Sepsis^non-BSI^) versus those without evidence of infection (No-sepsis). (**b**) Gene set enrichment analysis of the differentially expressed genes. All identified pathways are plotted. Source data including enriched genes and pathway P values (hypergeometric test) are provided in Supplementary Data 2b. Source data

**Extended Data Fig. 2:**
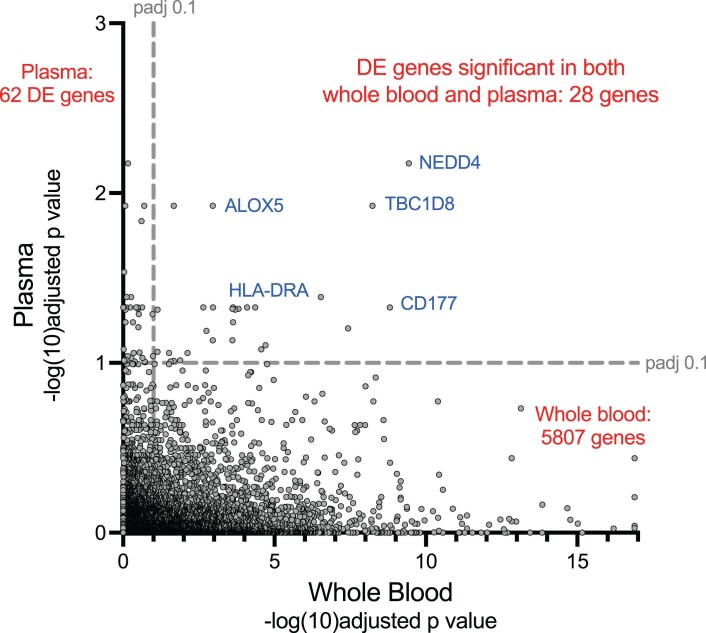
Overlap of significant genes in the differential expression analyses between the Sepsis and No-Sepsis groups for whole blood and plasma samples. Overlap of significant genes in the differential expression analyses between the Sepsis and No-Sepsis groups for whole blood and plasma samples. Scatter plot of -log10(adjusted p-value) for individual genes from the differential expression analyses comparing patients with microbiologically confirmed sepsis (Sepsis^BSI^ + Sepsis^non-BSI^) versus those without evidence of infection (No-sepsis), from whole blood (x-axis) and plasma (y-axis). P-values (two-sided) are derived from DESeq2 and incorporate Benjamini–Hochberg adjustment for multiple testing. Dashed grey lines indicate the threshold of adjusted p-value <0.1. Selected, significant, differentially expressed genes highlighted in blue. Source data

To test this, we sequenced RNA from patients with available plasma specimens matched to the whole-blood samples and obtained a median of 2.3 × 10^7^ (95% CI 2.2 × 10^7^–2.5 × 10^7^) reads per sample. Calculation of input RNA mass (Methods) demonstrated that samples with transcript counts below our quality control (QC) cut-off (<50,000) had a lower average input mass than those with sufficient counts (65.2 pg versus 85.8 pg, respectively, *P* < 0.0001, Supplementary Data 8). After filtering to retain samples with ≥50,000 transcripts (*n* = 138), we performed DE analysis to assess whether a biologically plausible signal could be observed between patients with sepsis (Sepsis^BSI^ and Sepsis^non-BSI^, *n* = 73) and those without (No-sepsis, *n* = 37), and found 62 genes at an adjusted *P* < 0.1 (Extended Data Fig. 1 and Supplementary Data 6), 28 of which were also significant in the whole-blood analysis (Extended Data Fig. 2). Remarkably, several of the top differentially expressed genes were previously reported sepsis biomarkers (for example, elevated *CD177*, suppressed *HLA-DRA*)^18–21^, suggesting a biologically relevant transcriptomic signature from plasma RNA (Fig. 2d and Supplementary Data 6).

We then asked whether a host transcriptional sepsis diagnostic classifier could be constructed using plasma RNA transcriptomic data by dividing the cohort into independent training (75% of data, *n* = 82) and validation groups (*n* = 28), and employing the same bSVM approach to select genes that most effectively distinguished Sepsis^BSI^ and Sepsis^non-BSI^ patients from No-sepsis patients. This approach yielded a classifier that achieved an average cross-validation AUC of 0.97 (s.d. 0.03) over 10 random splits within the training dataset (75% of data, *n* = 82). In the held-out validation set (25% of data, *n* = 28), an AUC of 0.77 was obtained. An AUC of 0.90 (s.d. 0.06) was obtained over 10 randomly generated validation sets (Fig. 2e and Supplementary Data 7).

### Detection of bacterial sepsis pathogens from plasma nucleic acid

**Fig. 3:**
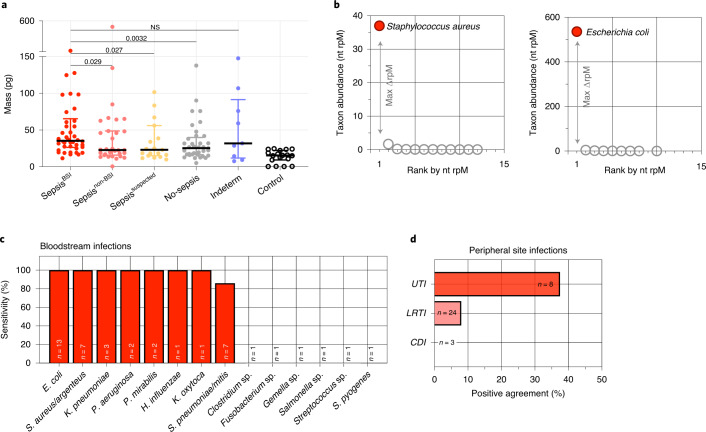
Plasma mNGS for detecting sepsis pathogens. **a**, Microbial plasma DNA mass differences between sepsis groups. Data are presented with a centre horizontal bar at the median, and error bars representing the interquartile ranges. Pairwise comparisons between groups were performed with a two-sided Mann–Whitney test. Sample sizes are as follows for each group: Sepsis^BSI^ *n* = 42, Sepsis^non-BSI^ *n* = 31, Sepsis^suspected^ *n* = 19, Indeterminate *n* = 9, No-sepsis *n* = 37, Control *n* = 18. Source data and *P* values for comparisons between samples, including water controls, are provided in Supplementary Data 8 and in the Source Data file. **b**, Graphical depiction of the RBM for sepsis pathogen detection from two different exemplary cases. The RBM identifies established pathogens with disproportionately high abundance compared with other commensal and environmental microbes in the sample. **c**, Concordance between plasma DNA mNGS for detecting bacterial pathogens in Sepsis^BSI^ patients and bacterial bloodstream infections compared to a reference standard of culture. **d**, Sensitivity of plasma nucleic acid mNGS for detecting pathogens in Sepsis^non-BSI^ patients with sepsis from non-bloodstream, peripheral sites of infection. LRTI = lower respiratory tract infection; UTI = urinary tract infection; CDI = *Clostridium difficile* infection. Clinical microbiology and metagenomics data are tabulated in Supplementary Data 9. Source data

We began microbial metagenomic analyses by assessing DNA microbial mass (Methods), which was significantly higher in Sepsis^BSI^ compared with other groups, except for the Indeterminate group. Microbial mass was significantly lower in the negative control water samples compared with each group (Fig. 3a and Supplementary Data 8). We next carried out bacterial pathogen detection using the IDseq pipeline^22^ for taxonomic alignment followed by a previously developed rules-based model (RBM)^14^ that identifies established sepsis pathogens overrepresented in mNGS data compared with less abundant commensal or contaminating microbes^14^ (Methods and Fig. 3b).

We then asked how well the metagenomic RBM pathogen predictions agreed with bacterial blood culture data. Polymicrobial blood cultures of ≥3 organisms were excluded (*n* = 2) given their unclear clinical relevance, leaving a total of 40 blood culture-positive cases available for comparison (Supplementary Data 9). Sensitivity versus blood culture as a reference standard was 83% and varied by pathogen, ranging from 0% (for example, *C. difficile*) to 100% (for example, *E. coli, S. aureus/argenteus*; Fig. 3c). Pathogens were called by the RBM in 10/37 (27%) patients in the No-sepsis group, equating to a specificity of 73%.

### Detection of sepsis pathogens from peripheral sites using plasma nucleic acid

Plasma DNA mNGS identified 2/25 (8%) culture-confirmed bacterial lower respiratory tract infection (LRTI) pathogens in the Sepsis^non-BSI^ group and 3/8 (38%) culture-confirmed bacterial urinary tract infection (UTI) pathogens (Fig. 3d and Supplementary Data 9). mNGS did not identify *C. difficile* in any of the three patients with severe colitis from this organism. Additional putative bacterial pathogens not detected by culture were detected in 8/73 (11%) patients with microbiologically confirmed sepsis (Supplementary Data 9).

### Identification of viral infections using host transcriptional profiling of RNA and whole blood

**Fig. 4:**
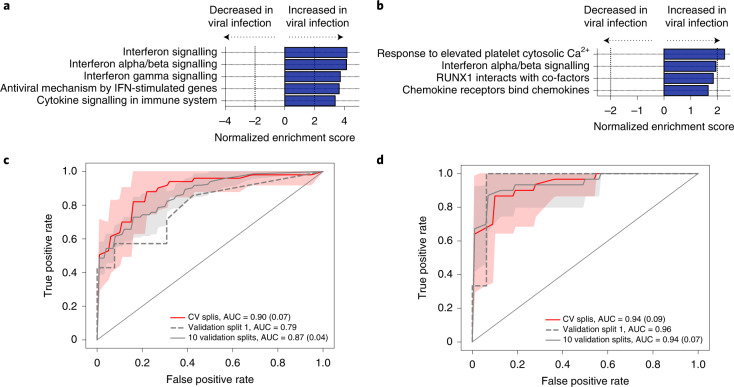
Detection of viral sepsis based on host gene expression. **a**, GSEA of differentially expressed genes from whole-blood RNA-seq (*n* = 129) demonstrating pathways enriched in patients with viral sepsis. The top five most significant pathways by *P* value (hypergeometric test) are plotted. Source data including enriched genes and pathway *P* values are provided in Supplementary Data 12a and in the Source Data file. **b**, GSEA of differentially expressed genes from plasma RNA-seq (*n* = 73) demonstrating pathways enriched in patients with viral sepsis. All identified pathways are plotted. Source data including enriched genes and pathway *P* values (hypergeometric test) are provided in Supplementary Data 12b and in the Source Data file. **c**, ROC curve demonstrating performance of the bSVM classifier for detecting viral sepsis from whole-blood RNA-seq (*n* = 129). The AUC and s.d. (in parentheses, when applicable) are listed for CV in the training set (red line: average over 10 random splits; red shaded area: ±1 s.d.), the held-out validation set (dashed grey line) and over 10 randomly generated validation sets (solid grey line: average; grey shaded area: ±1 s.d.). **d**, ROC curve demonstrating performance of the bSVM classifier for detecting viral sepsis from plasma RNA-seq (*n* = 73). The AUC and s.d. are listed for CV in the training set (red line: average over 10 random splits; red shaded area: ±1 s.d.), the held-out validation set (dashed grey line) and over 10 randomly generated validation sets (solid grey line: average; grey shaded area: ±1 s.d.). Source data

Only 1 of 13 (8%) respiratory viruses identified by clinical testing could be detected by plasma RNA mNGS (Supplementary Data 9). Recognizing that an alternative approach would be needed, we asked whether host response could instead be used to identify viral sepsis by carrying out differential gene expression analysis of patients with or without clinically confirmed viral sepsis within the Sepsis^BSI^ and Sepsis^non-BSI^ groups, using whole blood (Supplementary Data 10) or plasma (Supplementary Data 11) transcriptomic data. GSEA demonstrated that pathways related to interferon signalling and genes important for antiviral immunity were enriched in samples from patients with viral sepsis versus those with bacterial sepsis, in data derived from both whole blood (Fig. 4a and Supplementary Data 12a) and plasma (Fig. 4b and Supplementary Data 12b) datasets.

We then leveraged this host signature to build a secondary bSVM diagnostic classifier for viral sepsis selecting differentially expressed genes as potential predictors, which on whole-blood samples achieved an average cross-validation AUC of 0.90 (s.d. 0.07) over 10 random splits within the training dataset (75% of data, *n* = 96). In the held-out validation set (25% of data, *n* = 33), an AUC of 0.79 was obtained. An AUC of 0.87 (s.d. 0.04) was obtained over 10 randomly generated validation sets (Fig. 4c and Supplementary Data 13). Slightly better performance was obtained when building a classifier using plasma RNA-seq data, with an average cross-validation AUC of 0.94 (s.d. 0.09) over 10 random splits within the training dataset (75% of data, *n* = 54). In the held-out validation set (25% of data, *n* = 19), an AUC of 0.96 was obtained. An AUC of 0.94 (s.d. 0.07) was obtained over 10 randomly generated validation sets (Fig. 4d and Supplementary Data 14). Incorporation of the host-based viral sepsis classifier improved the sensitivity versus clinical respiratory viral PCR testing to 12/13 (92%) and predicted viral infection in one additional Sepsis^non-BSI^ patient who did not undergo viral PCR testing (Supplementary Data 15).

### Integrated host-microbe sepsis diagnostic model using plasma nucleic acid

**Fig. 5:**
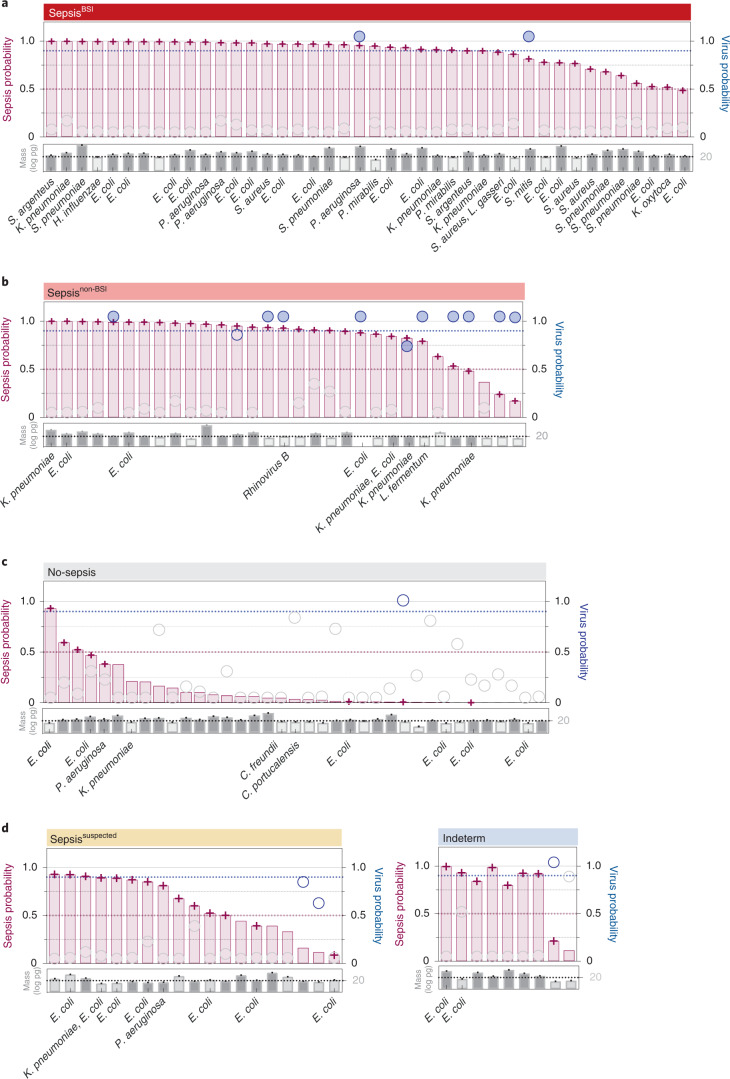
Integrated host-microbe model for sepsis diagnosis from plasma mNGS. **a**–**d**, Host criteria for positivity can be met by a sepsis transcriptomic classifier probability >0.5 (maroon bars, dotted line). Microbial criteria can be met on the basis of either: (1) detection of a pathogen by mNGS and a sample microbial mass (grey bars) >20 pg (dashed line), or (2) viral transcriptomic classifier probability >0.9 (blue circles, dotted line). Host and microbial metrics are highlighted for patients with sepsis due to bloodstream infections (Sepsis^BSI^) (**a**), peripheral infection (Sepsis^non-BSI^) (**b**), patients with non-infectious critical illness (No-sepsis) (**c**), patients with suspected sepsis but negative microbiological testing (Sepsis^suspected^) (**d**, left) and patients with indeterminate sepsis status (Indeterm) (**d**, right). Maroon cross, sepsis-positive based on model; blue circles, virus predicted from plasma RNA secondary viral host classifier; filled blue circles, virus also detected by clinical respiratory viral PCR. Cases with <20 pg microbial mass are indicated by lighter grey shading. Samples with mNGS-detected pathogens have the microbe(s) listed below the sample microbial mass. Raw values for plots and original training/test split assignments are tabulated in Supplementary Data 16 and provided in the Source Data file. Source data

Given the relative success of each independent host and pathogen model, we considered whether combining them could enhance diagnosis and potentially serve as a sepsis rule-out tool. To test this possibility, we developed a proof-of-concept integrated host + microbe model on the basis of simple rules. It returned a sepsis diagnosis on the basis of either host criteria: (host sepsis classifier probability >0.5) or microbial criteria: ((pathogen detected by RBM) AND (microbial mass >20 pg)) OR (host viral classifier probability >0.9). Applying these rules enabled detection of 42/42 (100%) cases in the Sepsis^BSI^ group and 30/31 (97%) cases in the Sepsis^non-BSI^ group, for an overall sensitivity of 72/73 (99%) (Fig. 5a,b). This proof-of-concept model yielded a specificity of 29/37 (78%) within the No-sepsis group (Fig. 5c and Supplementary Data 15).

### Application of the integrated model to suspected and indeterminate sepsis cases

Next, we asked whether patients with clinically adjudicated sepsis but negative in-hospital microbiologic testing (Sepsis^suspected^) would be predicted to have sepsis using the integrated host-microbe plasma mNGS model. Of the 19 patients, 14 (74%) were classified as having sepsis (Fig. 5d), 8 of which had a putative bacterial pathogen identified. Two additional patients had viral host classifier probabilities >0.5 but did not meet the threshold for sepsis-positivity in the integrated model. With respect to the indeterminate group, the integrated host + microbe model classified 8/9 (89%) as sepsis-positive (Fig. 5e and Supplementary Data 15). Of these, two had a putative bacterial pathogen identified and one had a putative viral infection identified by the viral host classifier.

### Comparison against clinical variable models for sepsis diagnosis

Lastly, we asked how host/microbe mNGS compared against sepsis diagnostic models derived exclusively from clinical metrics that would be available at the time of initial evaluation in the ED. We tested three different machine learning methods to distinguish Sepsis (Sepsis^BSI^ and Sepsis^non-BSI^) from No-sepsis patients, using 34 clinical variables as input (Supplementary Table 3). The data were split into training (75%) and validation (25%) sets, and model performance was evaluated on the latter. The greatest average AUC achieved was 0.62 (s.d. 0.04) using a random forest model (Supplementary Table 4). We then computed the AUC using the qSOFA score, a widely used clinical score for identifying patients with sepsis in the emergency department^15^. The qSOFA achieved an average AUC of 0.48 (s.d. 0.02).

## Discussion

Sepsis is defined as a dysregulated host response to infection^15^, yet existing diagnostics have focused exclusively on either detecting pathogens or assessing features of the infected host. Here we combined host transcriptional profiling with broad-range pathogen detection to accurately diagnose sepsis in critically ill patients upon hospital admission. Further, we demonstrate that an integrated host-microbe metagenomics approach can be performed on circulating RNA and DNA from plasma, a widely available clinical specimen type with previously unrecognized utility for host-based infectious disease diagnosis.

Identifying an aetiologic pathogen is critical for optimal treatment of sepsis. We found that concordance between pathogen detection by plasma mNGS and traditional bacterial blood culture varied by organism. For instance, mNGS sensitivity for detecting *S. aureus* and *E. coli*, two of the most globally important sepsis pathogens^5^, was 100%. In contrast, mNGS missed several important but less common sepsis pathogens, such as *S. pyogenes*. We noted that in all false-negative cases, the patients had received antibiotics before mNGS sample collection, and that research mNGS specimens collected up to 24 h after blood cultures may have resulted in lower concordance than if samples had been collected contemporaneously.

Several of the microbes missed by mNGS were organisms that in many contexts exist as commensals (for example, *Fusobacterium*, *Gemella* and *Streptococcus* species). It is unclear whether these organisms were truly aetiologic sepsis pathogens or commensals translocated to the blood in the setting of critical illness and incidentally identified in culture. With respect to non-BSI sepsis, our findings suggest that plasma mNGS may be most useful for identifying UTI-associated pathogens, although we also observed some utility for respiratory pathogen detection, in line with a previous report^23^. mNGS failed to detect *C. difficile* in any patients with colitis from this pathogen, although this is not surprising given that the organism is rarely associated with bacteremia^24^.

Within the No-sepsis group, 10/37 (27%) patients had a pathogen detected by mNGS. Notably, 9/10 (90%) pathogens were Gram-negative enteric organisms, which may reflect gastrointestinal translocation of microbes, a well-described phenomenon during critical illness^25^. In addition, all 10 of these patients had received antibiotics in the first day of study enrolment, so it is possible that sequences were derived from non-viable organisms unable to grow in culture.

Plasma RNA sequencing alone performed poorly for detecting sepsis-associated respiratory viruses. Incorporation of a host-based viral classifier, however, markedly improved detection of clinically confirmed viral LRTI. The viral classifier predicted previously unrecognized viral infections in three patients with sepsis who did not undergo viral PCR testing during their hospitalizations. Previous work has demonstrated that different viral species elicit distinct host transcriptional signatures in peripheral blood^26^, suggesting that future studies could extend the RNA host viral classifier to identify specific virus, such as influenza or SARS-CoV-2

In line with previous reports^13^, we found that viral sepsis has a unique host transcriptional signature characterized by expression of interferon and other signalling pathways. We also observed transcriptional differences based on whether sepsis was due to a bloodstream versus peripheral site infection, which was less expected, with Sepsis^BSI^ patients exhibiting lower expression of genes related to CD28 signalling and T-cell activation, and greater expression of genes related to antimicrobial peptides and defensins.

We found that detection of a pathogen alone was in many cases insufficient for sepsis diagnosis, but when combined with a host transcriptional profile, had promising diagnostic utility and potential as a tool for infection rule-out. In addition to defining host signatures of sepsis from whole blood, we also found biologically relevant host transcripts in plasma. This may have direct clinical applications given that plasma mNGS is increasingly being used in hospitals for pathogen detection in patients with sepsis and other infectious diseases, with turnaround times of ≤48 h.

Inappropriate antimicrobial use is a major challenge in the management of critical illness and is often driven by the inability to rule-out infection in patients with systemic inflammatory diseases. Indeed, we found that clinical variables alone, including the qSOFA score, were unable to accurately distinguish patients with sepsis from those with non-infectious critical illnesses at the time of initial evaluation in the ED. In contrast, our proof-of-concept assessment of the integrated host + microbe mNGS model demonstrated 99% sensitivity across patients with microbiologically confirmed sepsis, and 78% specificity within the No-sepsis group, which was composed almost entirely of patients meeting the clinical definition of systemic inflammatory response syndrome^16^.

Host/microbe mNGS may facilitate precision antimicrobial stewardship by discriminating sepsis from diverse types of non-infectious febrile inflammatory syndromes, ranging from autoimmune diseases to macrophage activation syndrome. We envision this assay being used at the time of ED presentation for all suspected sepsis patients, as an adjunct to blood cultures and other traditional microbiological testing.

Distinguishing true sepsis pathogens from environmental contaminants or human commensals is a challenge for both mNGS and traditional culture-based microbiologic methods. Concomitant assessment of a host-based metric offers an opportunity to determine whether the detected pathogen exists in the context of an immunological state consistent with infection. Considering this, host/microbe mNGS diagnostic classification could theoretically be more difficult in immunocompromised patients. Arguing against this, however, is previous work demonstrating accurate performance of a host/microbe mNGS pneumonia diagnostic in an ICU cohort with a 40% prevalence of immunocompromised individuals^14^.

Our study has several strengths, including the innovative use of plasma RNA transcriptomics for sepsis diagnosis, development of a sepsis diagnostic combining host and microbial mNGS data, detailed clinical phenotyping and a large prospective cohort of critically ill adults with systemic illnesses. It also has some limitations. First, as noted above, mNGS and blood cultures were performed on different samples collected at different times, so the observed concordance with clinical microbiological testing may be an underestimate. Second, several plasma samples had insufficient host transcripts to permit gene expression analyses, leading to a smaller sample size for the plasma versus the whole-blood cohorts. This limitation may be addressable in future studies by increasing the input amount of plasma RNA.

The host immune response during sepsis is dynamic, and thus the stage of infection at which gene expression is measured may influence accuracy of the classifier. While our study was cross-sectional in design, we attempted to control for this by sampling at a consistently early stage of critical illness, within the first 24 h of ICU admission. Lastly, because we did not have access to any other sepsis studies with either plasma gene expression data or paired host and microbial mNGS data from blood, additional studies in an independent cohort will be needed to validate these findings.

In conclusion, we report that combining host gene expression profiling and metagenomic pathogen detection from plasma nucleic acid enables accurate diagnosis of sepsis. Future studies are needed to validate and test the clinical impact of this culture-independent diagnostic approach.

## Methods

### Study design, clinical cohort and ethics statement

We conducted a prospective observational study of patients with acute critical illnesses, admitted from the ED to the ICU. We studied patients who were enrolled in the Early Assessment of Renal and Lung Injury (EARLI) cohort at the University of California, San Francisco (UCSF) or Zuckerberg San Francisco General Hospital (ZSFGH) between October 2010 and January 2018 (Supplementary Table 1). The study was approved by the UCSF Institutional Review Board (IRB) under protocol 10-02852, which granted a waiver of initial consent for blood sampling. Informed consent was subsequently obtained from patients or their surrogates for continued study participation^27,28^.

For the parent EARLI cohort, the inclusion criteria are: (1) age ≥18, (2) admission to the ICU from the ED and (3) enrolment in the ED or within the first 24 h of ICU admission. For this study, we selected patients for whom PAXgene whole-blood tubes and matched plasma samples from the time of enrolment were available. PAXgene tubes were collected on patients (enrolled in EARLI during the time period listed above) who were hypotensive and/or mechanically ventilated at the time of enrolment. The main exclusion criteria for the EARLI study are: (1) exclusively neurological, neurosurgical or trauma surgery admission, (2) goals of care decision for exclusively comfort measures, (3) known pregnancy, (4) legal status of prisoner and (5) anticipated ICU length of stay <24 h. Enrolment in EARLI began in October 2008 and continues. Study data were collected and managed using REDCap and Quesgen electronic data capture tools hosted at UCSF^29,30^.

### Sepsis adjudication

Clinical adjudication of sepsis groups was carried out by study team physicians (M.A., C.R.L., A.L., K.L., P.S., C.H., A.G., C.C., K.N.K., M.A.M.) using the sepsis-2 definition^31^ (≥2 SIRS criteria + suspected infection) and incorporating all available clinical and microbiologic data from the entire ICU admission, with blinding to mNGS results. Each patient was reviewed by at least four physicians. Disagreements were handled by discussion with the most senior physicians (C.S.C., M.A.M.) in the phenotyping panel. Patients were categorized into five subgroups on the basis of sepsis status (Fig. 1a): patients with clinically adjudicated sepsis and a bacterial culture-confirmed bloodstream infection (Sepsis^BSI^), sepsis due to a microbiologically confirmed primary infection at a peripheral site other than the bloodstream (Sepsis^non-BSI^), suspected sepsis with negative clinical microbiologic testing (Sepsis^suspected^), patients with no evidence of sepsis and a clear alternative explanation for their critical illness (No-sepsis), or patients of indeterminate status (Indeterm). Clinical and demographic features of patients are summarized in Supplementary Tables 1a,b and tabulated in Supplementary Data 16 and 17.

### Metagenomic sequencing

Following enrolment, whole blood and plasma were collected in PAXgene and EDTA tubes, respectively. Whole-blood PAXgene tubes (Qiagen, 762165) were processed and stored at −80 °C according to the manufacturer’s instructions, and plasma was frozen at −80 °C within 2 h. To evaluate host gene expression and detect microbes, RNA-seq was performed on the whole blood and plasma specimens, and DNA-seq was performed on plasma specimens. RNA was extracted from whole blood using the Qiagen RNeasy kit (Qiagen, 74004) and normalized to 10 ng total input per sample. Total plasma nucleic acid was extracted by first clarifying 300 μl of plasma via maximum-speed centrifugation for 5 min at 21,300 × *g*, and then employing the Zymo Pathogen Magbead kit (Zymo Research, R2145) on the supernatant following the manufacturer’s instructions. Total nucleic acid (10 ng) was subjected to DNA-seq using the NEBNext Ultra II DNA kit. Samples with at least 10 ng of remaining total nucleic acid were treated with DNAse (Qiagen) to recover RNA, and then subjected to RNA-seq library preparation using the NEBNext Ultra II RNA-seq kit (New England Biolabs, E7770S) as described below.

For RNA-seq library preparation, human cytosolic and mitochondrial ribosomal RNA and globin RNA were first depleted using FastSelect (Qiagen, 334385). For background contamination correction (see below) and to enable estimation of input microbial mass, we included negative water controls as well as positive controls (spike-in RNA standards from the External RNA Controls Consortium (ERCC); ThermoFisher, 4456740)^32^. RNA was then fragmented and subjected to library preparation using the NEBNext Ultra II RNA-seq kit (New England Biolabs, E7770S) according to the manufacturer’s instructions, with protocol optimization for a LabCyte Echo acoustic liquid handler^33^. Finished libraries underwent 146 nucleotide paired-end Illumina sequencing on an Illumina Novaseq 6000 instrument.

Index swapping can lead to read misassignment with Illumina sequencing. Dual indexing, that is, adding barcode index sequences on both ends of the molecule, reduces the rate at which this misassignment occurs by requiring concordance between the two barcode sequences. The frequency of index-swapped reads has been estimated to be more than 35× lower when using dual vs single indexing^34^. Because we used dual indexing and because the RBM for pathogen detection operates by only identifying pathogen sequences disproportionately abundant in a sample versus the other sequences, our methods would not be expected to be negatively influenced by index swapping, which would only be anticipated to misassign low abundance reads irrelevant to the RBM.

### Host differential expression and pathway analysis

Following demultiplexing, sequencing reads were aligned to an index of the human genome (NCBI GRC h38) plus ERCC RNA standards using STAR (version 2.6.1)^35^. Samples retained in the dataset had a total of at least 50,000 counts associated with protein coding genes. Differential expression analysis was performed using DESeq2 (ref. ^36^) and including covariates for age and gender. Significant genes were identified using an independent-hypothesis-weighted, Benjamini–Hochberg false discovery rate (FDR) < 0.1^37,38^. We generated heat maps of the top 50 differentially expressed genes by absolute log_2_-fold change. To evaluate signalling pathways from gene expression data, we employed gene set enrichment analysis using WebGestalt^39^ on all ranked differentially expressed genes with *P* value <0.1. Significant pathways and upstream regulators were defined as those with a gene set *P* value <0.05.

### Pathogen detection

Detection of microbes leveraged the open-source IDseq pipeline (v3.7, https://czid.org/), which incorporates subtractive alignment of the human genome (NCBI GRC h38) using STAR^35^ (v2.5.3), quality and complexity filtering, and subsequent removal of cloning vectors and phiX phage using Bowtie2 (v2.3.4)^22^. The identities of the remaining microbial reads were determined by querying the NCBI nucleotide (NT) database using GSNAP-L^22,40^ in the final steps of the IDseq pipeline. After background correction (see below), retained non-viral taxonomic alignments in each sample were aggregated at the genus level and sorted in descending order by abundance measured in reads per million (rpM), independently for each sample. A previously validated RBM^14^ was then utilized to identify disproportionately abundant bacteria and fungi in each sample, and flag them as pathogens. The RBM, originally developed to identify pathogens from respiratory mNGS data, detects outlier organisms within a sample by identifying the greatest gap in abundance between the top 15 sequentially ranked microbes in each sample. All microbes present in a reference index of established pathogens above this gap are then called by the RBM.

We adapted the original RBM specifically for sepsis pathogen detection, in which outlier organisms are sometimes present in low abundance, by incorporating a sepsis (as opposed to a respiratory) pathogen reference index (Supplementary Data 18) and requiring that the species called by the RBM be both present in the reference index and detected at an abundance >1 rpM. Given the potential for respiratory viruses to cause sepsis, the RBM also identified human pathogenic respiratory viruses derived from a reference list of LRTI pathogens^14^, present in the plasma RNA-seq data at an abundance of >1 rpM. Sensitivity and specificity were calculated on the basis of detection of reference index sepsis pathogens in each of the sepsis adjudication groups.

The reference index (Supplementary Data 18) was established a priori and no data from the enrolled patients were used to inform the distinction between pathogens and commensals. The index consisted of the most prevalent bloodstream infection pathogens reported by both the National Healthcare Safety Network (NHSN)^41^ and a recent multicentre surveillance study of healthcare-associated infections^42^. These studies reported multiple species of *Bacteroides*, *Candida*, *Citrobacter*, *Enterobacter*, *Enterococcus*, *Klebsiella*, *Lactobacillus*, *Morganella*, *Prevotella*, *Proteus*, *Serratia*, *Stenotrophomonas* and *Streptococcus* as common sepsis pathogens, and thus the reference index contains all species within these genera, yielding >1,000 total species detectable by the model based on current NCBI taxonomy.

### Identification and mitigation of environmental contaminants

Negative control samples consisting of only double-distilled water (*n* = 24) were processed alongside plasma DNA samples, which were sequenced in a single batch. Negative control samples enabled estimation of the number of background reads expected for each taxon^43^. A previously developed negative binomial model^43^ (https://github.com/czbiohub/idseqr/) was employed to identify taxa with NT sequencing alignments present at an abundance significantly greater compared with negative water controls. This was done by modelling the number of background reads as a negative binomial distribution, with mean and dispersion fitted on the negative controls. For each taxon, we estimated the mean parameter of the negative binomial by averaging the read counts across all negative controls. We estimated a single dispersion parameter across all taxa, using the functions glm.nb() and theta.md() from the R package MASS^44^ (v7.3-51). Taxa that achieved an adjusted *P* < 0.01 (Benjamini and Hochberg multiple test correction) were carried forward to the above-described RBM for pathogen detection.

### Microbial mass calculations

Microbial mass was calculated on the basis of the ratio of microbial reads in each sample to total reads aligning to the External RNA Controls Consortium (ERCC) RNA standards spiked into each sample^32^. The following equation was utilized for this calculation: (ERCC input mass)/(microbial input mass) = (ERCC reads)/(microbial reads), where the ERCC input mass was 25 pg.

### Host transcriptional classifiers for viral sepsis diagnosis

To build classifiers that differentiated patients with sepsis (Sepsis^BSI^, Sepsis^non-BSI^) from those with non-infectious critical illness (No-sepsis), and distinguished viral from non-viral sepsis, we built a support vector machine (SVM)-based classifier^45^ with the scikit-learn^46^ (v0.23.2) library in Python (v3.8.3). We tested several machine learning approaches (bagged SVM, random forest and gradient boosted trees) and selected a bSVM classifier with a linear kernel based on best performance (Supplementary Table 2). Each classifier used a bootstrapped set of samples and a random subset of features.

We evaluated samples with ≥50,000 plasma gene counts and genes with more than 20% non-zero counts in that sample subset. Only differentially expressed genes, identified using DESeq2 (v1.28.1) in the training set, were considered as potential predictors and included in machine learning models, with FDR thresholds of 0.1 (whole blood), 0.2 (plasma, viral) and 0.3 (plasma, sepsis) chosen on the basis of cross validation. Age and sex were included as covariates in the models. We used *Z*-score-scaled transformed (variance stabilizing transformation) gene counts. To train the model, 75% of the data was selected and the rest was used as a held-out set to test the final model. The training set was subsequently randomly split ten times for cross validation, using 75% of each as intermediate training sets, and the remaining 25% as their associated testing sets.

On each one of those intermediate training sets, we carried out feature selection and parameter optimization using nested 5-fold cross-validations. We optimized three parameters: the regularization parameter, the maximum number of features considered for each classifier and the total number of classifiers to use for bagging. For each parameter’s optimization fold, a recursive feature elimination strategy was adopted, dropping 10% of the remaining least important features at each iteration. A bSVM classifier with default parameters was built at each iteration. We defined feature importance as the average squared weight across all estimators. To maximize interpretability, we restricted the maximum number of predictors to 100 genes.

We estimated model performances using the AUC values. To obtain a single set of features, we fitted a model, using the aforementioned strategy, to the initial training set. This model was then tested on the held-out set to obtain a final performance value and a single set of predictors.

### Comparison of plasma mNGS against clinician-ordered testing

Clinical microbiological testing was carried out on the basis of decisions from the primary medical team during the patient’s hospital admission at the UCSF and the ZSFGH clinical microbiology laboratories. Tests utilized included bacterial culture from blood, lower respiratory tract and urine, which were carried out in the clinical microbiology laboratories at each hospital as previously described^14^. Clinical testing for viral respiratory pathogens was performed from nasopharyngeal swabs and/or bronchioalveolar lavage using the Luminex XTag multiplex viral PCR assay. Polymicrobial blood cultures with ≥3 bacteria (*n* = 2) were excluded from pathogen concordance given their unclear clinical relevance and potential that some organisms reflected contamination.

### Integrated host + microbe sepsis diagnosis and rule-out model

We developed a simple integrated host + microbe model that returned a sepsis diagnosis on the basis of either host criteria (host sepsis classifier probability >0.5) or microbial criteria ((pathogen detected by RBM) AND (microbial mass >20 pg)) OR (host viral classifier probability >0.9). Combined metrics (Supplementary Data 16) including sepsis assignment based on this model are depicted in Fig. 5. Sensitivity was calculated in the Sepsis^BSI^ and Sepsis^non-BSI^ groups, and specificity in the No-sepsis group.

### Clinical variable models for sepsis diagnosis

We tested the ability of clinical variables (Supplementary Table 3) available at the time of initial patient assessment to predict sepsis using three machine learning methods. These included SVM using the e1071 package^47^ v1.7, random forest using the randomForest package^48^ v4.7 and regularized logistic regression using the glmnet package^49^ v4.1 in R v4.2.0^50^. Specifically, we built models to classify Sepsis (Sepsis^BSI^ and Sepsis^non-BSI^) versus No-sepsis using 34 clinical variables that were available at the time of ED evaluation. The data were split into training (75%) and test (25%) sets, and model performance (AUC) was evaluated on the test set. This was repeated for a total of 10 randomized splits, with the AUC computed at each iteration. AUC was also computed for the qSOFA score (systolic blood pressure <100 mmHg, respiratory rate >22 breaths per minute, Glasgow coma scale <13). Results are tabulated in (Supplementary Table 4).

### Statistics and reproducibility

Statistical tests utilized for each analysis are described in the figure legends and in further detail in each respective Methods section. The numbers of patient samples analysed for each comparison are indicated in the figure legends. Data were generated from single sequencing runs without technical replicates.

### Reporting summary

Further information on research design is available in the Nature Research Reporting Summary linked to this article.

## Supplementary information

Supplementary Information Supplementary Tables 1–4, descriptions of Supplementary Data files, and references.

Reporting Summary

Supplementary Data Supplementary Data 1–18.

## Data Availability

The processed genecount data are available from the National Center for Biotechnology Information Gene Expression Omnibus database under accession code GSE189403. The raw sequencing data are protected due to data privacy restrictions from the IRB protocol governing patient enrolment, which protects the release of raw genetic sequencing data from those patients enrolled under a waiver of consent. To honour this, researchers who wish to obtain raw fastq files for the purposes of independently generating gene counts can contact the corresponding author (chaz.langelier@ucsf.edu) and request to be added to the IRB protocol. The raw fastq files with microbial sequencing reads are available from the Sequence Read Archive under BioProject IDs: PRJNA782906 and PRJNA782908. Source data are provided with this paper.

## Source data

Source Data Fig. 2 Results from gene set enrichment analysis of differentially expressed genes (whole blood RNA-seq) between patients with microbiologically confirmed sepsis (Sepsis^BSI^ and Sepsis^non-BSI^) and those with non-infectious critical illnesses (No-sepsis).
Source Data Fig. 3 Mass (pg) of microbial DNA in each sample.
Source Data Fig. 4 Results from gene set enrichment analysis of differentially expressed genes between patients with viral versus non-viral causes of sepsis among the Sepsis^BSI^ and Sepsis^non-BSI^ patients. **a**, Data from whole blood RNA-seq. **b**, Data from plasma RNA-seq.
Source Data Fig. 5 Per-patient probability values from the sepsis transcriptomic classifier and viral transcriptomic classifiers. Microbial mass and pathogens detected by the rules-based model.
Source Data Extended Data Fig. 1 Results from gene set enrichment analysis of differentially expressed genes between patients with microbiologically confirmed sepsis (Sepsis^BSI^ and Sepsis^non-BSI^) and those with non-infectious critical illnesses (No-sepsis). Data from plasma RNA-seq.
Source Data Extended Data Fig. 2 Differentially expressed genes between patients with microbiologically confirmed (Sepsis^BSI^ and Sepsis^non-BSI^) and those with non-infectious critical illnesses (No-sepsis), from whole blood RNA-seq and plasma. Raw values and log_10_-transformed values tabulated.

## Code availability

Code for the differential expression, classifier development and RBM can be found at https://github.com/lucile-n/plasma_classifiers.
