# Supplementary material for: Integrated host-microbe plasma metagenomics for sepsis diagnosis in a prospective cohort of critically ill adults
Source: Nat Microbiol. 2022 Oct 20;7(11):1805–16. doi: 10.1038/s41564-022-01237-2 (PMC9613463; doi:10.1038/s41564-022-01237-2)
Supplement: Supplementary file 1 — Supplementary Tables 1–4, descriptions of Supplementary Data files, and references. [file 41564_2022_1237_MOESM1_ESM.docx]

**Supplementary Information, Table of Contents**

Supplementary Tables page 2

Supplementary Table 1a page 2

Supplementary Table 1b page 3

Supplementary Table 2 page 4

Supplementary Table 3 page 5

Supplementary Table 4 page 6

Supplementary Data File Descriptions page 7

Supplementary References page 11

**Supplementary Table 1a.** Summary of clinical and demographic features of patients evaluated in whole blood gene expression analyses (n=221). These include patients with microbiologically confirmed sepsis (Sepsis^BSI^ and Sepsis^non-BSI^) and those with non-infectious critical illnesses (No-Sepsis). Source data are tabulated in (Supplementary Data 16).

| **Whole Blood** |  | **Sepsis^BSI^**  (n=60) | **Sepsis^non-BSI^** (n=69) | **No-Sepsis** (n=92) | **Sepsis**  vs **No-Sepsis**  P value* |
| --- | --- | --- | --- | --- | --- |
| Age  (median, Q1-Q3/%) |  | 63.5 (50.8-73.3) | 68 (58-80) | 65.5 (54-74.5) | 0.51 |
| Gender (median, %) | Male | 41 (68.3%) | 40 (58%) | 53 (57.6%) | 0.49 |
|  | Female | 19 (31.7%) | 28 (40.6%) | 39 (42.4%) |  |
|  | Transgender | 0 (0.0%) | 1 (1.4%) | 0 (0.0%) |  |
| Race (n, %) | Caucasian | 20 (33.3%) | 29 (42%) | 37 (40.2%) | 0.63 |
|  | Asian | 17 (28.3%) | 21 (30.4%) | 24 (26.1%) |  |
|  | African American | 10 (16.7%) | 11 (15.9%) | 17 (18.5%) |  |
|  | Other | 12 (20.0%) | 5 (7.2%) | 14 (15.2%) |  |
|  | Unknown | 0 (0.0%) | 3 (4.3%) | 0 (0.0%) |  |
|  | Native American | 1 (1.7%) | 0 (0.0%) | 0 (0.0%) |  |
| Ethnicity (n, %) | Non-LatinX | 51 (85.0%) | 60 (87%) | 79 (85.9%) | 0.76 |
|  | LatinX | 9 (15.0%) | 6 (8.7%) | 12 (13.0%) |  |
|  | NA | 0 (0.0%) | 3 (4.3%) | 1 (1.1%) |  |
| Temp Max  (median, Q1-Q3%) |  | 38.1  (37.1-38.9) | 37.6  (37-38.4) | 37.3  (36.1-37.9) | 1.20e-5 |
| WBC Max  (median, Q1-Q3%) |  | 15.5  (10.1-23.8) | 13.9  (9.5-19.2) | 13.15  (9.5-18.9) | 0.24 |
| APACHEIII  (median, Q1-Q3%) |  | 69 (53-85) | 50 (46-66) | 64 (50-78) | 0.17 |
| SIRS | 4 | 39 (65.0%) | 26 (37.7%) | 27 (29.3%) | 0.01 |
|  | 3 | 16 (26.7%) | 30 (43.5%) | 42 (45.7%) |  |
|  | 2 | 5 (8.3%) | 12 (17.4%) | 22 (23.9%) |  |
|  | 1 | 0 (0.0%) | 0 (0.0%) | 1 (1.1%) |  |
| Bacterial infection^‡^ |  | 60 (100.0%) | 50 (72.5%) | 0 (0.0%) | 0 |
| Viral +/- Bacterial infection^‡^ |  | 3 (5.0%) | 21 (30.4%) | 0 (0.0%) | 3.72e-4 |
| 28-day mortality (n, %) |  | 23 (38.3%) | 15 (21.7%) | 32 (34.8%) | 0.49 |
| Intubated (n, %) |  | 47 (78.3%) | 59 (85.5%) | 86 (93.5%) | 0.02 |
| Vasopressors (n, %) |  | 54 (90.0%) | 51 (73.9%) | 59 (64.1%) | 6.22e-3 |
| Immunocompromised^#^ (n, %) |  | 7 (11.7%) | 6 (8.7%) | 6 (6.5%) | 0.49 |
| Antibiotics^+^ (n, %) |  | 59 (98.3%) | 66 (95.7%) | 73 (79.3%) | 6.65e-5 |

*Sepsis^BSI^ + Sepsis^non-BSI^ vs No-Sepsis P value calculated by two-sided Mann-Whitney (continuous) or chi-squared (categorical)

^‡^Based on clinical microbiology testing

^#^Immunocompromise was defined as: history of solid organ transplantation, bone marrow transplantation, HIV/AIDS with CD4 < 200, leukemia or other hematologic malignancy, autoimmune inflammatory disease, or primary immunodeficiency.

^+^Antibiotics administered on or before the first day of study enrollment.

**Supplementary Table 1b.** Summary of clinical and demographic features of patients with plasma RNA-seq data, evaluated in all analyses (n=138). All sepsis adjudication groups represented. Source data are tabulated in (Supplementary Data 17).

| **Plasma** |  | **Sepsis^BSI^ (n=42)**  Median/n  (Q1-Q3/%) | **Sepsis^non-BSI^ (n=31)**  Median/n (Q1-Q3/%) | **No-Sepsis** **(n=37)**  Median/n  (Q1-Q3/%) | P value*  Sepsis^BSI+non-BSI^  v No-Sepsis | **Sepsis^suspected^** **(n=19)**  Median/n  (Q1-Q3/%) | **Indeterm (n=9)**  Median/n  (Q1-Q3/%) | Overall  P value^†^ |
| --- | --- | --- | --- | --- | --- | --- | --- | --- |
| Age  (median, Q1-Q3/%) |  | 64 (51-72) | 69 (58-79) | 66 (55-79) | 0.45 | 69 (55-80) | 72 (63-80) | 0.12 |
| Gender  (median, %) | Male | 28 (66.7%) | 17 (54.8%) | 21 (56.8%) | 0.77 | 9 (47.4%) | 3 (33.3%) | 0.24 |
|  | Female | 14 (33.3%) | 14 (45.2%) | 16 (43.2%) |  | 9 (47.4%) | 6 (66.7%) |  |
|  | Transgender | 0 (0.0%) | 0 (0.0%) | 0 (0.0%) |  | 1 (5.3%) | 0 (0.0%) |  |
| Race (n, %) | Caucasian | 13 (31%) | 12 (38.7%) | 14 (37.8%) | 0.86 | 4 (21.1%) | 7 (77.8%) | 0.50 |
|  | Asian | 12 (28.6%) | 10 (32.3%) | 13 (35.1%) |  | 9 (47.4%) | 1 (11.1%) |  |
|  | African American | 8 (19%) | 6 (19.4%) | 7 (18.9%) |  | 3 (15.8%) | 1 (11.1%) |  |
|  | Other | 8 (19%) | 2 (6.5%) | 3 (8.1%) |  | 3 (15.8%) | 0 (0.0%) |  |
|  | Native American | 1 (2.4%) | 0 (0.0%) | 0 (0.0%) |  | 0 (0.0%) | 0 (0.0%) |  |
|  | Unknown | 0 (0.0%) | 1 (3.2%) | 0 (0.0%) |  | 0 (0.0%) | 0 (0.0%) |  |
| Ethnicity (n, %) | Non-LatinX | 36 (85.7%) | 28 (90.3%) | 35 (94.6%) | 0.48 | 16 (84.2%) | 8 (88.9%) | 0.62 |
|  | LatinX | 6 (14.3%) | 2 (6.5%) | 2 (5.4%) |  | 3 (15.8%) | 1 (11.1%) |  |
|  | NA | 0 (0.0%) | 1 (3.2%) | 0 (0.0%) |  | 0 (0.0%) | 0 (0.0%) |  |
| Temp Max  (median, Q1-Q3/%) |  | 38.1  (37.1-38.9) | 37.8  (37.1-38.5) | 37.1  (36.1-38) | 2.4e-3 | 37  (36.6-38.2) | 35.8  (34.9-37.2) | 6.0e-4 |
| WBC Max  (median, Q1-Q3/%) |  | 16.1  (6.5-26.6) | 17.4  (11.2-22.8) | 13  (9.9-20.6) | 0.40 | 19.3  (12.8-26.1) | 15.3  (9.7-19.7) | 0.60 |
| APACHEIII  (median, Q1-Q3/%) |  | 72.5 (52-85) | 54 (46-69) | 72 (57-81) | 0.20 | 70 (61-89) | 72 (65-93) | 0.04 |
| SIRS | 4 | 28 (66.7%) | 13 (41.9%) | 15 (40.5%) | 0.19 | 12 (63.2%) | 5 (55.6%) | 0.52 |
|  | 3 | 11 (26.2%) | 15 (48.4%) | 15 (40.5%) |  | 5 (26.3%) | 3 (33.3%) |  |
|  | 2 | 3 (7.1%) | 3 (9.7%) | 6 (16.2%) |  | 2 (10.5%) | 1 (11.1%) |  |
|  | 1 | 0 (0.0%) | 0 (0.0%) | 1 (2.7%) |  | 0 (0.0%) | 0 (0.0%) |  |
| Bacterial infection^‡^ |  | 42 (100.0%) | 24 (77.4%) | 0 (0.0%) | <2.2e-16 | 0 (0.0%) | 0 (0.0%) | <2.2e-16 |
| Viral +/- Bacterial infection^‡^ |  | 2 (4.8%) | 11 (35.5%) | 0 (0.0%) | 0.02 | 0 (0.0%) | 0 (0.0%) | 1.5e-6 |
| 28-day mortality (n, %) |  | 20 (47.6%) | 8 (25.8%) | 18 (48.6%) | 0.41 | 11 (57.9%) | 7 (77.8%) | 0.04 |
| Intubated (n, %) |  | 32 (76.2%) | 28 (90.3%) | 35 (94.6%) | 0.13 | 18 (94.7%) | 9 (100%) | 0.05 |
| Vasopressors (n, %) |  | 40 (95.2%) | 23 (74.2%) | 31 (83.8%) | 0.95 | 18 (94.7%) | 8 (88.9%) | 0.08 |
| Immunocompromised^#^ (n, %) |  | 6 (14.3%) | 5 (16.1%) | 4 (10.8%) | 0.75 | 5 (26.3%) | 2 (22.2%) | 0.63 |
| Antibiotics^+^ (n, %) |  | 41 (97.6%) | 31 (100.0%) | 31 (83.8%) | 9.3e-3 | 19 (100.0%) | 8 (88.9%) | 0.02 |

*Two-sided Mann-Whitney (continuous) /chi-squared (categorical) ^†^Two-side Kruskal-Wallis (continuous) / chi-squared (categorical)

^‡^Based on clinical microbiology testing

^#^Immunocompromise was defined as: history of solid organ transplantation, bone marrow transplantation, HIV/AIDS with CD4 < 200, leukemia or other hematologic malignancy, autoimmune inflammatory disease, or primary immunodeficiency.

^+^Antibiotics administered on or before the first day of study enrollment.

**Supplementary Table 2. Comparison of machine learning models for host-based sepsis classification.** Area under the receiver operator characteristic curve (AUC) for three different machine learning models assessed for classifier construction. The AUC for cross-validation in the training set (standard deviation in parentheses) is listed first, and the AUC for the first validation split is listed second, after the vertical bar. The bagged support vector machine (bSVM) model performed best overall.

| **Classifier** | **Bagged Support Vector Machine** | **Random**  **Forest** | **Gradient**  **Boosted Tree** |
| --- | --- | --- | --- |
| Whole blood - sepsis | 0.81 (0.05) \| 0.82 | 0.80 (0.06) \| 0.86 | 0.79 (0.05) \| 0.84 |
| Plasma - sepsis | 0.97 (0.03) \| 0.77 | 0.76 (0.05) \| 0.79 | 0.70 (0.09) \| 0.82 |
| Whole blood - viral | 0.90 (0.07) \| 0.79 | 0.83 (0.08) \| 0.75 | 0.78 (0.07) \| 0.69 |
| Plasma - viral | 0.94 (0.09) \| 0.96 | 0.77 (0.09) \| 0.81 | 0.72 (0.19) \| 0.60 |

**Supplementary Table 3.** Clinical variables used for classifier construction. Fever: > 38C, anemia: hemoglobin < 7, thrombocytopenia: platelets < 50, acute renal failure: creatinine > 2.0.

| Anemia | Temp Max | Creatinine Max |
| --- | --- | --- |
| Hyperkalemia | Fever | Creatinine Min |
| Hypokalemia | Temp Min | Acute Renal Failure |
| Hypernatremia | WBC Max | Platelets Min |
| Hyponatremia | WBC Min | Thrombocytopenia |
| Hypercalcemia | HR Max | Requirement for Intubation |
| Hypocalcemia | HR Min | Glasgow Coma Scale |
| Hyperthyroid | RR Max | Immunocompromise |
| Hypothyroid | RR Min | Chest Pain |
| Adrenal Insufficiency | SIRS total | Volume Overload |
| Hyperglycemia | SBP Min | Creatinine Max |
| Hypoglycemia | SBP Max | Creatinine Min |

**Supplementary Table 4.** Average classifier AUC over 10 iterations of training and test from different machine learning classifiers to distinguish Sepsis from No-Sepsis patients using clinical features alone. qSOFA score positive for sepsis if systolic blood pressure < 100 mmHg, respiratory rate > 22 breaths/minute and Glasgow Coma Scale < 13.

| **Method** | **AUC, mean (std)** |
| --- | --- |
| Support vector machine | 0.57 (0.04) |
| Random forest | 0.62 (0.04) |
| Regularized logistic regression | 0.57 (0.07) |
| qSOFA score | 0.48 (0.02) |

**Supplementary Data Files**

Supplementary Data 1. Differentially expressed genes (adjusted P value < 0.1) between patients with microbiologically confirmed (Sepsis^BSI^ and Sepsis^non-BSI^) and those with non-infectious critical illnesses (No-Sepsis), from whole blood RNA-seq.

Supplementary Data 2. Gene set enrichment analysis of differentially expressed genes between patients with microbiologically confirmed sepsis (Sepsis^BSI^ and Sepsis^non-BSI^) and those with non-infectious critical illnesses (No-Sepsis). Data from a) whole blood RNA-seq and b) plasma RNA-seq. The top 10 positively and negatively enriched pathways by P value (hypergeometric test) are included in table.

Supplementary Data 3. Differentially expressed genes (adjusted P value < 0.1) between patients with sepsis due to bloodstream infections (Sepsis^BSI^) versus peripheral infections (Sepsis^non-BSI^). Data from whole blood RNA-seq.

Supplementary Data 4. Gene set enrichment analysis of differentially expressed genes between patients with sepsis due to bloodstream infections (Sepsis^BSI^) versus peripheral infections (Sepsis^non-BSI^). Data from whole blood RNA-seq. The top 10 positively and negatively enriched pathways by P value (hypergeometric test) are included in table.

Supplementary Data 5. a) Area under the receiver operating characteristic curve (AUC) values for 10 independent training set models for a whole blood gene expression support vector machine classifier to distinguish patients with microbiologically confirmed (Sepsis^BSI^ and Sepsis^non-BSI^) from those with non-infectious critical illnesses (No-Sepsis). b) Composite list of all genes selected by each classifier model.

Supplementary Data 6. Differentially expressed genes (adjusted P value < 0.1) between patients with microbiologically confirmed (Sepsis^BSI^ and Sepsis^non-BSI^) and those with non-infectious critical illnesses (No-Sepsis), from plasma RNA-seq.

Supplementary Data 7. a) AUC values for 10 independent training set models for a plasma gene expression support vector machine classifier to distinguish patients with microbiologically confirmed (Sepsis^BSI^ and Sepsis^non-BSI^) from those with non-infectious critical illnesses (No-Sepsis). b) Composite list of all genes selected by each classifier model.

Supplementary Data 8. Mass (pg) of microbial DNA in each sample, calculated based on spiked-in 25 pg reverse-transcribed ERCC positive controls.

Supplementary Data 9. Sepsis pathogens detected by standard of care clinical microbiology versus plasma mNGS, using the rules-based model.

Supplementary Data 10. Differentially expressed genes (adjusted P value < 0.1) between patients with microbiologically confirmed viral sepsis and those with non-viral sepsis (Sepsis^BSI^ and Sepsis^non-BSI^ groups), from whole blood RNA-seq.

Supplementary Data 11. Differentially expressed genes (adjusted P value < 0.1) between patients with microbiologically confirmed viral sepsis and those with non-viral sepsis (Sepsis^BSI^ and Sepsis^non-BSI^ groups), from plasma RNA-seq.

Supplementary Data 12. Gene set enrichment analysis of differentially expressed genes between patients with viral versus non-viral causes of sepsis amongst the Sepsis^BSI^ and Sepsis^non-BSI^ patients. a) Data from whole blood RNA-seq. b) Data from plasma RNA-seq.

Supplementary Data 13. a) AUC values for 10 independent training set models for a whole blood gene expression support vector machine classifier to distinguish patients with microbiologically confirmed viral versus non-viral sepsis (Sepsis^BSI^ and Sepsis^non-BSI^), from whole blood RNA-seq. b) Composite list of all genes selected by each classifier model.

Supplementary Data 14. a) AUC values for 10 independent training set models for a plasma gene expression support vector machine classifier to distinguish patients with microbiologically confirmed viral versus non-viral sepsis (Sepsis^BSI^ and Sepsis^non-BSI^), from plasma RNA-seq. b) Composite list of all genes selected by each classifier model.

Supplementary Data 15. Complete integrated host-microbe mNGS dataset. This includes: per-sample classifier predictions for all patients with plasma sequencing data (n=138), including the sepsis diagnostic classifier and the viral sepsis classifier; pathogens detected by clinical diagnostics and by mNGS; and microbial mass per sample.

Supplementary Data 16. Clinical and demographic features of patients evaluated in whole blood gene expression analyses only (n=221). These include patients with microbiologically confirmed sepsis (Sepsis^BSI^ and Sepsis^non-BSI^) and those with (No-Sepsis).

Supplementary Data 17. Clinical and demographic features of patients with plasma RNA-seq data, evaluated in all analyses (n=138). All sepsis adjudication groups represented.

Supplementary Data 18. Reference index of established sepsis pathogens derived from the top 20 most prevalent sepsis pathogens reported by both the US CDC/ National Healthcare Safety Network^1^ and a point prevalence survey of healthcare-associated infections^2^.

**Supplementary References**

1. Weiner-Lastinger, L. M. *et al.* Antimicrobial-resistant pathogens associated with adult healthcare-associated infections: Summary of data reported to the National Healthcare Safety Network, 2015–2017. *Infect. Control Hosp. Epidemiol.* **41**, 1–18 (2020).

2. Magill, S. S. *et al.* Changes in Prevalence of Health Care–Associated Infections in U.S. Hospitals. *New England Journal of Medicine* **379**, 1732–1744 (2018).
