# Supplementary material for: Integrated host-microbe plasma metagenomics for sepsis diagnosis in a prospective cohort of critically ill adults
Source: Nat Microbiol. 2022 Oct 20;7(11):1805–16. doi: 10.1038/s41564-022-01237-2 (PMC9613463; doi:10.1038/s41564-022-01237-2)
Supplement: Supplementary file 2 — Reporting Summary [file 41564_2022_1237_MOESM2_ESM.pdf]

nature portfolio

Corresponding author(s): Charles Langelier

Last updated by author(s): Aug 7, 2022

Reporting Summary

Nature Portfolio wishes to improve the reproducibility of the work that we publish. This form provides structure for consistency and transparency in reporting. For further information on Nature Portfolio policies, see our Editorial Policies and the Editorial Policy Checklist.

Statistics

For all statistical analyses, confirm that the following items are present in the figure legend, table legend, main text, or Methods section.

n/a	Confirmed	
[ ]	[x]	The exact sample size (*n*) for each experimental group/condition, given as a discrete number and unit of measurement
[ ]	[x]	A statement on whether measurements were taken from distinct samples or whether the same sample was measured repeatedly
[ ]	[x]	The statistical test(s) used AND whether they are one- or two-sided<br>*Only common tests should be described solely by name; describe more complex techniques in the Methods section.*
[ ]	[x]	A description of all covariates tested
[ ]	[x]	A description of any assumptions or corrections, such as tests of normality and adjustment for multiple comparisons
[ ]	[x]	A full description of the statistical parameters including central tendency (e.g. means) or other basic estimates (e.g. regression coefficient) AND variation (e.g. standard deviation) or associated estimates of uncertainty (e.g. confidence intervals)
[ ]	[x]	For null hypothesis testing, the test statistic (e.g. *F*, *t*, *r*) with confidence intervals, effect sizes, degrees of freedom and *P* value noted<br>*Give P values as exact values whenever suitable.*
[x]	[ ]	For Bayesian analysis, information on the choice of priors and Markov chain Monte Carlo settings
[x]	[ ]	For hierarchical and complex designs, identification of the appropriate level for tests and full reporting of outcomes
[x]	[ ]	Estimates of effect sizes (e.g. Cohen's *d*, Pearson's *r*), indicating how they were calculated

Our web collection on statistics for biologists contains articles on many of the points above.

Software and code

Policy information about availability of computer code

Data collection	Clinical data were collected and stored using QuesGen and REDCap databases
Data analysis	Bulk RNAseq and differential expression:<br>Following demultiplexing, sequencing reads were aligned with STAR to an index consisting of all transcripts associated with human protein coding genes (ENSEMBL v. 99), cytosolic and mitochondrial ribosomal RNA sequences, and the sequences of ERCC RNA standards. Samples retained in the dataset had a total of at least 50,000 counts associated with transcripts of protein coding genes. Differential expression analysis was performed using DESeq2 and including covariates for age and gender. Significant genes were identified using an independent-hypothesis-weighted, Benjamini-Hochberg false discovery rate (FDR) < 0.1.<br><br>Classifier construction:<br>To build gene expression classifiers that differentiated patients with sepsis from those with non-infectious critical illness, and distinguished viral from non-viral sepsis, we built a Support Vector Machine (SVM)-based classifier with the scikit-learn (v0.23.2) library in Python (v3.8.3). To build clinical variable classifiers we tested three different machine learning methods. These included SVM using the e1071 package v1.7, random forest using the randomForest package v4.7 and regularized logistic regression using the glmnetpackage v4.1 in R v4.2.0.<br><br>Pathogen detection:<br>Detection of microbes leveraged the open-source IDseq pipeline v3.7 (https://czid.org/) which incorporates subtractive alignment of the human genome (NCBI GRC h38) using STAR (v2.5.3), quality and complexity filtering, and subsequent removal of cloning vectors and phiX phage using Bowtie2 (v2.3.4). The identities of the remaining microbial reads are determined by querying the NCBI nucleotide (NT) database using GSNAP-L in the final steps of the IDseq pipeline.<br><br>Background correction:<br>Negative control samples enabled estimation of the number of background reads expected for each taxon. A previously developed negative

binomial model (https://github.com/czbiohub/idseqr/) was employed to identify taxa with NT sequencing alignments present at an abundance significantly greater compared to negative water controls. This was done by modeling the number of background reads as a negative binomial distribution, with mean and dispersion fitted on the negative controls. For each taxon, we estimated the mean parameter of the negative binomial by averaging the read counts across all negative controls. We estimated a single dispersion parameter across all taxa, using the functions glm.nb() and theta.md() from the R package MASS (v7.3-51).

Code availability:
Code for differential gene expression, classifier development and pathogen detection can be found at: (https://github.com/lucile-n/plasma_classifiers).

For manuscripts utilizing custom algorithms or software that are central to the research but not yet described in published literature, software must be made available to editors and reviewers. We strongly encourage code deposition in a community repository (e.g. GitHub). See the Nature Portfolio guidelines for submitting code & software for further information.

Data

Policy information about availability of data

All manuscripts must include a data availability statement. This statement should provide the following information, where applicable:

- Accession codes, unique identifiers, or web links for publicly available datasets
- A description of any restrictions on data availability
- For clinical datasets or third party data, please ensure that the statement adheres to our policy

Study data were collected and managed using REDCap and Quesgen electronic data capture tools hosted at UCSF. Source data are provided with this paper. The processed genecount data are available from the National Center for Biotechnology Information Gene Expression Omnibus database under accession code GSE189403. The raw sequencing data are protected due to data privacy restrictions from the IRB protocol governing patient enrollment in this study, which protect the release of raw genetic sequencing data from those patients enrolled under a waiver of consent. To honor this, researchers who wish to obtain raw fastq files for the explicit purpose of independently generating genecounts for assessing gene expression can contact the corresponding author (chaz.langelier@ucsf.edu) and request to be added to the IRB protocol. Requests will be addressed within a timeframe of two weeks. The raw fastq files with microbial sequencing reads are available from the Sequence Read Archive under BioProject IDs: PRJNA782906 and PRJNA782908.

Field-specific reporting

Please select the one below that is the best fit for your research. If you are not sure, read the appropriate sections before making your selection.

- [x] Life sciences
- [ ] Behavioural & social sciences
- [ ] Ecological, evolutionary & environmental sciences

For a reference copy of the document with all sections, see nature.com/documents/nr-reporting-summary-flat.pdf

Life sciences study design

All studies must disclose on these points even when the disclosure is negative.

Sample size	Samples were selected from an observational cohort. We used the RNASeqPower package for R to calculate the power of differential expression analysis, and determined that we had greater than 99% to detect a 2-fold change in expression at an FDR < 0.1 in our primary analysis.
Data exclusions	The main exclusion criteria for the cohort were: 1) exclusively neurological, neurosurgical, or trauma surgery admission, 2) goals of care decision for exclusively comfort measures, 3) known pregnancy, 4) legal status of prisoner, and 5) anticipated ICU length of stay < 24 hours. Enrollment in EARLI began in 10/2008 and continues.
Replication	All analyses were performed in a single cohort of patients. We have made a concerted attempt to clearly indicate the number of patients analyzed in each comparator group (Sepis-BSI, Sepsis-non-BSI, Sepsis-suspected, No Sepsis) in the manuscript and figure legends. This is the first publicly available host/microbe sequencing dataset of sepsis patients, and there is therefore no dataset available for a replication analysis.
Randomization	N/A - observational study
Blinding	Investigators were blinded to group allocation during data collection. Investigators were blinded to any information about gene expression or metagenomic sequencing prior to chart review for sepsis adjudication. The sequencing and alignment pipeline did not have any information about the subject diagnosis.

Reporting for specific materials, systems and methods

We require information from authors about some types of materials, experimental systems and methods used in many studies. Here, indicate whether each material, system or method listed is relevant to your study. If you are not sure if a list item applies to your research, read the appropriate section before selecting a response.

Materials & experimental systems

n/a	Involved in the study	
☒	☐	Antibodies
☒	☐	Eukaryotic cell lines
☒	☐	Palaeontology and archaeology
☒	☐	Animals and other organisms
☐	☒	Human research participants
☐	☒	Clinical data
☒	☐	Dual use research of concern

Methods

n/a	Involved in the study	
☒	☐	ChIP-seq
☒	☐	Flow cytometry
☒	☐	MRI-based neuroimaging

Human research participants

Policy information about studies involving human research participants

Population characteristics

We conducted a prospective observational study of adults with acute critical illnesses admitted from the ED to the ICU at the University of California, San Francisco (UCSF) or Zuckerberg San Francisco General Hospital between 10/2010 and 01/2018. We studied patients who were enrolled in the longstanding Early Assessment of Renal and Lung Injury (EARLI) cohort. Detailed demographic and clinical characteristics of the cohort and analyzed patient groups are provided in Supplementary Table 1.

Recruitment

We would like to note that in the manuscript, we reference 2 prior studies which describe recruitment in detail:

Auriemma, C. L. et al. Acute respiratory distress syndrome-attributable mortality in critically ill patients with sepsis. Intensive Care Med 46, 1222–1231 (2020).

Agrawal, A. et al. Plasma angiopoietin-2 predicts the onset of acute lung injury in critically ill patients. Am J Respir Crit Care Med 187, 736–742 (2013).

We would also like to provide a more comprehensive description here:

If a patient met inclusion criteria for the EARLI cohort, then a study coordinator or physician obtained written informed consent for enrollment from the patient or their surrogate. Patients or their surrogates were provided with detailed written and verbal information about the goals of the study, the data and specimens that would be collected, and the potential risks to the subject. Patients and their surrogates were also informed that there would be no benefit to them from being enrolled in the study and that they may withdraw informed consent at any time during the course of the study. All questions were answered, and informed consent documented by obtaining the signature of the patient or their surrogate on the consent document.

Many critically ill patients are unconscious at the time of intensive care unit (ICU) admission due to their underlying illness and/or are endotracheally intubated for airway management or acute respiratory failure. The patients who are not unconscious are often in pain and may have acute delirium due to critical illness and/or medications. For these reasons, many subjects are unable to provide informed consent at the time of enrollment. Because this study could not practically be done otherwise and was deemed to be minimal risk by the UCSF IRB, if a patient was unable and a surrogate was not available to provide consent, patients were enrolled with waiver of initial consent, including the collection of biological samples.

Specifically, for subjects who were unable to provide informed consent at the time of enrollment, our study team was permitted to collect biological samples as well as clinical data from the medical record obtained prior to consent. Surrogate consent was vigorously pursued for all patients; moreover, each patient was regularly examined to determine if and when s/he was able to consent for him/herself, and the nursing and ICU staff were contacted daily for information about surrogates' availability. For patients whose surrogates provided informed consent, follow-up consent was subsequently obtained from the patient if they survived their acute illness and regained the ability to consent. For subjects who died prior to the consent being obtained, a full waiver of consent was approved by the UCSF IRB for both cohort studies.

Lack of a surrogate to provide consent is common in critically ill patients. To address this, the UCSF IRB also approved a full waiver of consent for subjects who remained unable to provide informed consent and had no contactable surrogate identified within 28 days. Before utilizing this waiver, we made and documented at least three separate attempts to identify and contact the patient or surrogate over a month-long period. No personally identifiable information has been included as part of this manuscript for any enrolled patients.

Lastly, we would like to note that patients with more severe disease (e.g., mechanical ventilation, hypotension) were preferentially selected for inclusion, and thus our study population may not be representative of every patient transferred from the ED to ICU.

Ethics oversight

We conducted a prospective observational study of patients with acute critical illnesses admitted from the ED to the ICU. We studied patients who were enrolled in the Early Assessment of Renal and Lung Injury (EARLI) cohort at the University of California, San Francisco (UCSF) or Zuckerberg San Francisco General Hospital between 10/2010 and 01/2018 (Supplementary Table 1). The study was approved by the UCSF Institutional Review Board (IRB) under protocol 10-02852, which granted a waiver of initial consent for blood sampling. Informed consent was subsequently obtained from patients or their surrogates for continued study participation, as previously described above and in the following references:

Auriemma, C. L. et al. Acute respiratory distress syndrome-attributable mortality in critically ill patients with sepsis. Intensive Care Med 46, 1222–1231 (2020).
Agrawal, A. et al. Plasma angiopoietin-2 predicts the onset of acute lung injury in critically ill patients. Am J Respir Crit Care Med 187, 736–742 (2013).

Note that full information on the approval of the study protocol must also be provided in the manuscript.

Clinical data

Policy information about clinical studies

All manuscripts should comply with the ICMJE guidelines for publication of clinical research and a completed CONSORT checklist must be included with all submissions.

Clinical trial registration	N/A
Study protocol	N/A
Data collection	We studied patients who were enrolled in the Early Assessment of Renal and Lung Injury (EARLI) cohort at the University of California, San Francisco (UCSF) or Zuckerberg San Francisco General Hospital between 10/2010 and 01/2018.
Outcomes	The primary outcome was diagnosis of sepsis using host +/- microbial metagenomics. Secondary outcomes included pathogen detection by metagenomics and host-based identification of viral sepsis.
